# Post-transcriptional regulation of BRCA1 through its coding sequence by the miR-15/107 group of miRNAs

**DOI:** 10.3389/fgene.2015.00242

**Published:** 2015-07-24

**Authors:** Kevin Quann, Yi Jing, Isidore Rigoutsos

**Affiliations:** Computational Medicine Center, Sidney Kimmel Medical College, Thomas Jefferson University, PhiladelphiaPA, USA

**Keywords:** microRNAs, miRNAs, non-coding RNA, BRCA1, post-transcriptional regulation, DNA damage repair

## Abstract

MicroRNAs (miRNAs) are important post-transcriptional regulators of gene expression that act by degrading their RNA targets or by repressing the translation of messenger RNAs (mRNAs). Initially thought to primarily target the 3′ untranslated region (3′UTR) of mRNAs, miRNAs have since been shown to also target the 5′UTR and coding sequence (CDS). In this work, we focus on the post-transcriptional regulation of the *BRCA1* gene, a major tumor suppressor and regulator of double-stranded break DNA repair and show that its mRNA is targeted by many members of the miR-15/107 group at a site located within the CDS. Ectopic expression of these miRNAs across a panel of nine cell lines demonstrated widespread suppression of BRCA1 mRNA levels. Additionally, by cloning a putative target site from BRCA1’s amino acid CDS into a luciferase reporter plasmid we confirmed the direct interaction of these miRNAs with this BRCA1 target. We also examined the relationship between ectopic expression of these targeting miRNAs and BRCA1 protein levels in immortalized pancreatic epithelium (hTERT-HPNE), colorectal adenocarcinoma (HCT-116) and pancreatic adenocarcinoma (MIA PaCa-2) cell lines and found protein abundance to be variably regulated in a cell-type specific manner that was not necessarily concordant with mRNA transcript availability. Our findings reveal a previously unrecognized aspect of BRCA1’s miRNA-mediated post-transcriptional regulation, namely the targeting of its amino acid coding region by the miR-15/107 group of miRNAs. The resulting regulation is apparently complex and cell-specific, an observation that may have implications for BRCA1-mediated DNA repair across tissue types.

## Introduction

MicroRNAs (miRNAs) are short non-coding RNAs, approximately 20–24 nts in length, which are widely regarded as key post-transcriptional regulators of gene expression. As part of the RNA-induced silencing complex (RISC) that comprises the Argonaute (Ago) proteins, miRNAs act on their targets through RNA degradation or inhibition of mRNA translation (He and Hannon, 2004; Filipowicz et al., 2008). To date, most of the studied miRNA target sites, also known as MREs, have been within the 3′UTR of target mRNA transcripts and characterized by miRNA-MRE heteroduplexes with Watson–Crick base pairing within the seed region, which comprises nts 2–7 from the 5′ end of the miRNA. The targeting of 3′UTR regions and the formation of Watson–Crick base pairs in the seed region are the hallmarks of what has been known as the *standard* model of miRNA targeting (Brodersen and Voinnet, 2009).

Early work by us and others has led to the identification of many functional MREs that are located within the CDS of mRNAs. Since then, the topic of CDS targeting by miRNAs has been attracting increasing attention in several disease contexts (Duursma et al., 2008; Forman et al., 2008; Lal et al., 2008; Shen et al., 2008b; Tay et al., 2008; Rigoutsos, 2009). In addition to CDS targets, several groups including ours have also reported miRNA targets in the 5′UTR of mRNAs (Lee et al., 2009; Grey et al., 2010; Zhou and Rigoutsos, 2014). Several of these non-3′UTR targets as well as many 3′UTR targets happened to contain various combinations of bulges and G:U wobbles in the seed region, instead of the Watson–Crick base pairs of the standard model (Ha et al., 1996; Lal et al., 2008; Tay et al., 2008; Chi et al., 2012; Loeb et al., 2012). The possibility that miRNA targeting beyond the 3′UTR could be frequent has since been emphasized with the advent of HITS-CLIP, PAR-CLIP, CLASH, and related methods that helped generate evidence to this effect in multiple cell types (Chi et al., 2009; Hafner et al., 2010; Helwak et al., 2013; Clark et al., 2014).

BRCA1 is a critical regulator of genomic integrity through its function as a mediator of homology directed repair (HDR) of double-stranded DNA breaks. As part of the DNA damage response (DDR), this process is essential for the maintenance of error-free chromosomal content during cell division and safeguards tissues from the accumulation of oncogenic or otherwise pathogenic mutations (Roy et al., 2012; Rosen, 2013). Recent therapeutic strategies aimed at inhibiting DNA repair pathways have met with success for BRCA1-deficient tumors due to effects of synthetic lethality (Helleday et al., 2008). Several BRCA1 studies to date have uncovered miRNA targets within the 3′UTR of the mRNA, however, we are not aware of any reports of miRNA targets in the BRCA1 coding region (Chang and Sharan, 2012).

The miR-15/107 group is a large family that comprises 10 miRNAs: miR-103a, miR-107, miR-15a, miR-15b, miR-16, miR-195, miR-424, miR-497, miR-503, and miR-646 (Finnerty et al., 2010). All 10 miRNAs share a common seed-region sequence (AGCAGC): miR-103 and miR-107 are an exception in that in these two miRNAs AGCAGC spans positions 1–6, instead of 2–7. The members of the group are conserved across chordate species, with miR-195, miR-424, miR-503, and miR-646 being exclusive to mammals (Kozomara and Griffiths-Jones, 2014). MiRNAs from this group are expressed in a wide variety of tissues and given that many of their validated targets are involved in cell cycle, metabolism, and angiogenesis, it follows that dysregulation of these miRNAs is a hallmark of many disease states (Aqeilan et al., 2010; Finnerty et al., 2010; Li et al., 2011; Furuta et al., 2013). In fact, every cell type studied to date is known to express one or more of the group’s miRNAs (Finnerty et al., 2010) further highlighting this miRNA group’s ubiquitous regulation of many cellular biochemical processes. Interestingly, recent experimental profiling of miR-103 and miR-107 revealed their frequent participation in targeting interactions with the CDS of mRNAs (Nelson et al., 2011).

In this report, we describe and analyze the direct molecular coupling between miRNAs of the miR-15/107 group and BRCA1 and in particular, focus on an MRE that is located in the coding region of BRCA1 mRNA. Given the extensive length of the BRCA1 transcript it is reasonable to assume that unrecognized MREs may exist in it and in particular in its long coding region. Along these lines, we sought to investigate the potential for this gene to be regulated by members of the ubiquitous miR-15/107 group through previously unrecognized non-standard interactions across a variety of tissues. Moreover, we examined the potential for tissue-specific variation secondary to complex miRNA–mRNA target networks using panels of cancerous and non-cancerous cell lines as model systems.

## Materials and Methods

### Cell Culture and Reagents

Human cell lines hTERT-HPNE, MCF7, MDA-MB-231, MDA-MB-468, HT-29, HCT-116, MIA PaCa-2, and PANC-1 were obtained from the American Type Culture Collection (ATCC, Manassas, VA, USA). 293T cells were obtained from Thermo-Fisher Scientific (Pittsburgh, PA, USA). 293T, hTERT-HPNE, MCF7, MDA-MB-231, MDA-MB-468, MIA PaCa-2, and PANC-1 cells were propagated in DMEM (Mediatech, Manassas, VA, USA) supplemented with 10% FBS and 1% penicillin/streptomycin (Life Technologies, Carlsbad, CA, USA). HT-29 and HCT-116 cells were propagated in McCoy’s 5A medium (Sigma–Aldrich, St. Louis, MO, USA) supplemented with 10% FBS and 1% penicillin/streptomycin. Precursor mature miRNAs to hsa-miR-15a-5p, hsa-miR-16-5p, hsa-miR-103a-3p, hsa-miR-107-5p, hsa-miR-195-5p, hsa-miR-424-5p, hsa-miR-497-5p, hsa-miR-503-5p, and anti-miRs anti-miR-15a-5p, anti-miR-16-5p, anti-miR-103a-3p, anti-miR-107-5p, anti-miR-195-5p, anti-miR-424-5p, anti-miR-497-5p, anti-miR-503-5p, and negative controls for miR-precursors and anti-miRs were obtained from Life Technologies.

### Transient Transfection of miRNA Precursors and Antisense miRNA Plasmids

Cells were plated in 6-well plates at a density of 0.5 × 10^6^ and transfected 24 h later with either 50 nM of miRNA precursor or 1 μg of psi-Check2 plasmid containing antisense miRNA sequences inserted into the multiple cloning site using Lipofectamine RNAiMax or Lipofectamine 2000 reagent, respectively, as per manufacturer’s instructions (Life Technologies). Forty-eight hours following transfection, cells were harvested for downstream analysis.

### Quantitative Reverse Transcriptase PCR

Total RNA was obtained from cells using Trizol reagent and reverse-transcribed to cDNA using oligo(dT) primers and Superscript III reverse transcriptase as per manufacturer’s instructions (Life Technologies). TaqMan qRT-PCR probes for BRCA1 and ACTB were used in conjunction with 10 ng of template cDNA in Fast Advanced Master Mix according to manufacturer’s instructions (Life Technologies). All samples were amplified on an Applied Biosystems Step One Plus thermocycler (Life Technologies). Relative gene expression levels were calculated by the ΔΔCT method, with normalization to ACTB.

### Luciferase Validation of miRNA Targets

Oligonucleotides corresponding to the predicted target site or antisense miRNA sequences were synthesized and included flanking XhoI and NotI restriction sites (Life Technologies). After annealing to respective antisense oligonucleotides, target fragments were double-digested with the appropriate restriction enzymes (New England Biolabs, Ipswich, MA, USA) and cloned into the 3′UTR of *Renilla* luciferase within the psi-Check2 dual luciferase reporter plasmid (Promega, Madison, WI, USA). 293T cells were plated at a concentration of 5,000 cells per well in 96-well plates and transfected the following day with 100 ng of psi-Check2 reporter plasmid and 50 nM of miRNA precursor per well using Lipofectamine 2000 as per manufacturer’s instructions (Life Technologies). Forty-eight hours following transfection luciferase levels were measured using a Dual Luciferase Reporter Kit (Promega). For analysis, target *Renilla* luciferase activity was normalized to control firefly luciferase values.

### Western Immunoblotting

Cells harvested 72 h post transfection were resuspended in RIPA lysis buffer (10 mM Tris-Cl, 1 mM EDTA, 1% Triton X-100, 0.1% sodium deoxycholate, 0.1% SDS, 140 mM NaCl) containing protease inhibitors (Thermo Fisher, Waltham, MA, USA) and cleared of cellular debris by centrifugation at 10,000 × *g* for 10 min. Lysate protein concentrations were obtained by BCA assay (Thermo Fisher) with equal amounts subjected to SDS-PAGE and electrophoretic transfer to nitrocellulose membranes. All membranes were blocked in TBST with 5% BSA prior to overnight incubation with primary antibody, washing, and subsequent incubation with appropriate HRP-conjugated secondary antibody. All membranes were developed using ECL chemiluminescent reagents (Thermo Fisher) and exposed on an ImageQuant LAS4000 imaging system (GE Healthcare). Primary antibodies include anti-BRCA1 (D54A8, Cell Signaling, Danvers, MA, USA) and anti-β-actin (8H10D10, Cell Signaling). Secondary antibodies include HRP-goat-anti-mouse (Cell Signaling) and HRP-Goat-anti-rabbit (Life Technologies).

### Statistical Analyses

All data derived from qRT-PCR and luciferase expression experiments are expressed as mean ± SD with a sample size of *n* = 3 for each group. Statistical analyses between experimental and control groups were performed with unpaired two-tailed Student’s *t*-test assuming equal variances (*degrees of freedom* = *n_control_* + *n_experimental_* – 2 = 4). Statistical significance was assumed at *P* < 0.05.

## Results

### The miR-15/107 Group is Predicted to Target the Coding Sequence of BRCA1 mRNA

**FIGURE 1:**
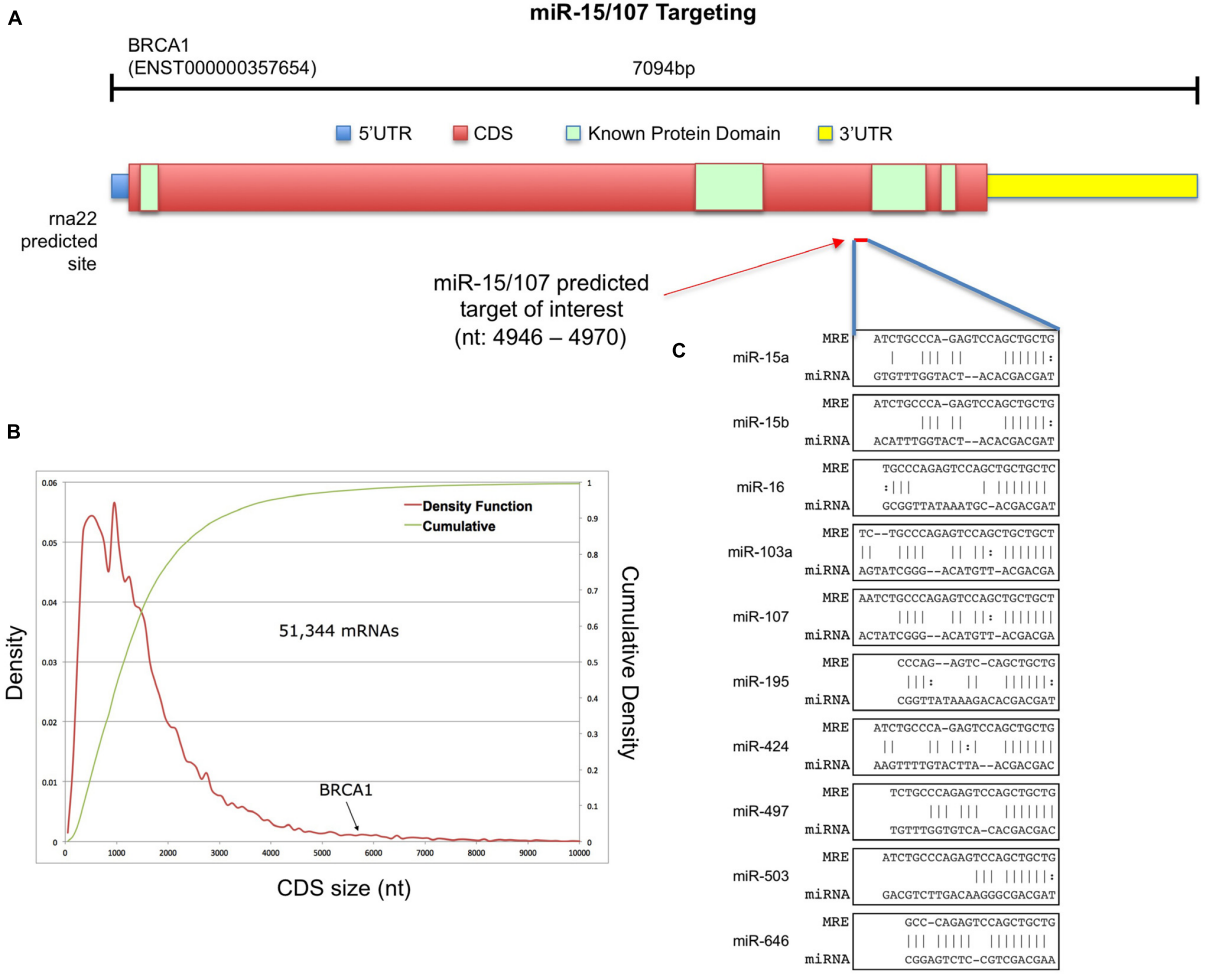
**Putative miR-15/107 targets within the BRCA1 CDS. (A)** Schematic representation of protein-coding BRCA1 transcript. **(B)** Density function and cumulative distribution of CDS length across all human coding mRNAs. **(C)** The rna22-predicted target site for the miR-15/107 group together with the corresponding miRNA:mRNA heteroduplexes.

BRCA1, a critical gene responsible for the maintenance of genomic integrity that is commonly inactivated or dysregulated in a number neoplastic processes, possesses a long CDS. At more than 5.5 kb (78% of the mRNA length), the amino acid coding region of BRCA1’s mRNAs places it among the top 2% of all protein-coding transcripts by CDS size (**Figures 1A,B**). We reasoned that such a long CDS might harbor miRNA targets that would not be predicted by focusing on standard, 3′UTR-centered interactions. Among candidate post-transcriptional regulators of BRCA1 mRNA abundance, we focused on the miR-15/107 group for three reasons: most of the group’s members are conserved in primates and rodents; the targets of two of its members, miR-103 and miR-107, have been shown to be enriched in coding region MREs and every known cell type expresses at least one of the group’s members (Nelson et al., 2011). Using the rna22 target prediction algorithm (Miranda et al., 2006) we sought candidate target MREs for the miR-15/107 group and identified regions along the full-length transcript of BRCA1 (ENST000000357654) that contained at least one MRE specific to a miR-15/107 miRNA. One predicted target site within the CDS which features the codon sequences for three consecutive alanine residues (GCTGCTGCT) just upstream of BRCA1’s conserved BRCT1 regions. This putative site is very well conserved in primates and several non-primate mammals (Supplementary Figure S1) and was predicted to form several heteroduplexes with the miRNAs of the miR-15/107 group characterized by Watson–Crick base-pairing in the seed-region (**Figure 1C**).

### The miR-15/107 Group Negatively Regulates BRCA1 mRNA Abundance

**FIGURE 2:**
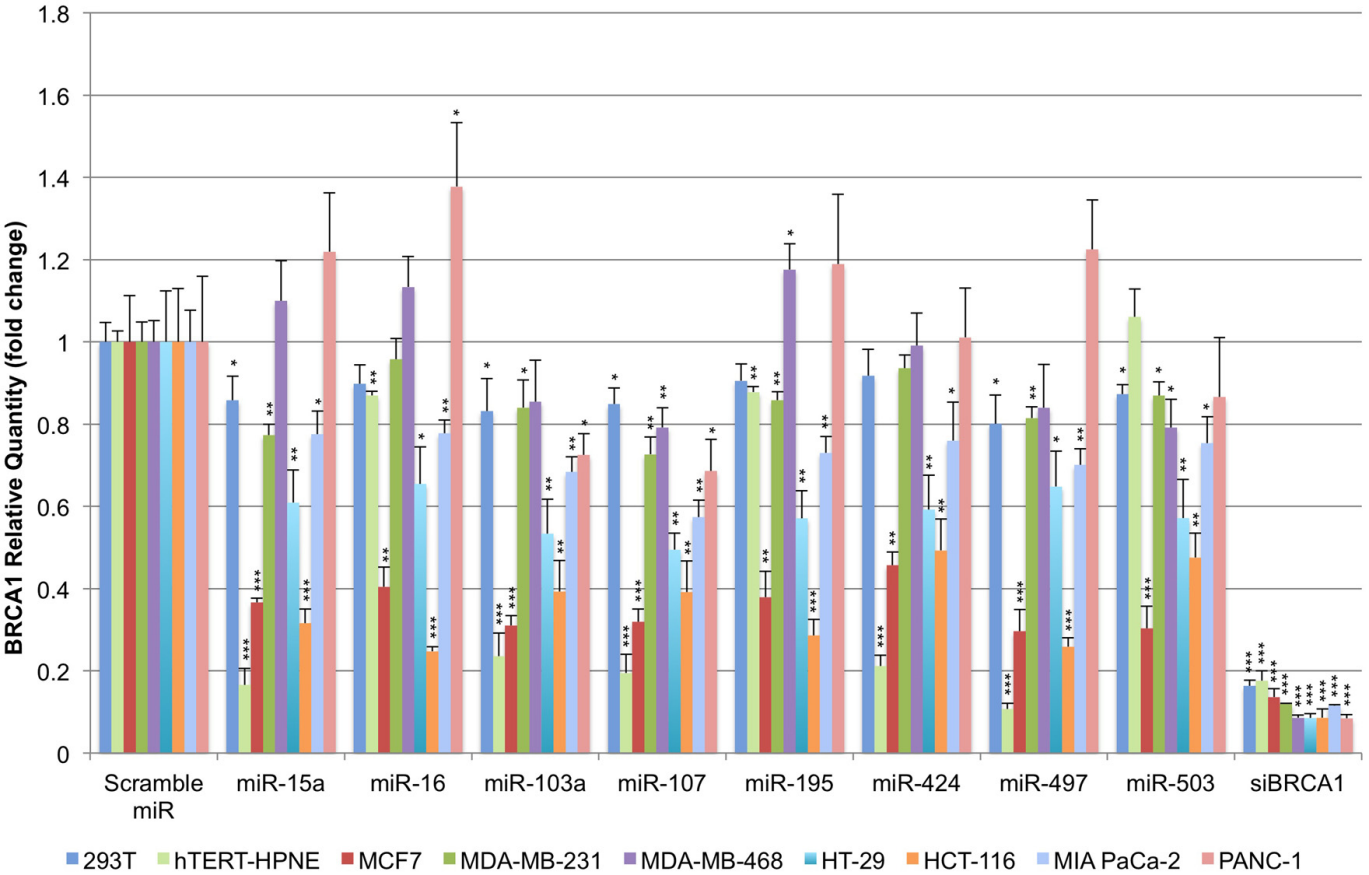
**Suppression of BRCA1 mRNA by miR-15/107 miRNAs.** Cell lines transfected with miR-15/107 miRNA precursors show a significant decrease in the amount of available BRCA1 transcript 48 h post-transfection as detected by qRT-PCR. ^∗^*P* < 0.05, ^∗∗^*P* < 0.01, ^∗∗∗^*P* < 0.001 compared to control scramble miR by Student’s *t*-test, *n* = 3 in each group.

Given the ubiquitous presence of miR-15/107 miRNAs in cells we sought to evaluate how well their interaction with BRCA1 persists across model cell lines. We selected nine BRCA1-competent model cell lines for this purpose and transiently transfected them with the respective miRNA precursors. The nine cell lines were: 293T (embryonic kidney epithelium), hTERT-HPNE (normal immortalized pancreatic epithelium) MCF7 (ER+ breast carcinoma), MDA-MB-231 and MDA-MB-468 (metastatic breast carcinoma), HT-29 and HCT-116 (invasive colorectal cancer), and MIA PaCa-2 and PANC-1 (pancreatic adenocarcinoma). We selected a panel of 8 out of 10 miR-15/107 miRNAs that, based on the literature, were the most representative of this group and found to be widely expressed in many tissues: miR-15a, miR-16, miR-103a, miR-107, miR-195, miR-424, miR-497, and miR-503 (Landgraf et al., 2007). Using quantitative real-time PCR, we observed that 48 h post-transfection, miR-15/107 precursors were able to suppress BRCA1 transcript in a cell-dependent manner (**Figure 2**). MCF7 and HCT-116 cells responded the most consistently and robustly across the eight tested miR-15/107 precursors, with an average BRCA1 mRNA suppression at 35% of control transfected cells (*P* < 0.001). HT-29 cell demonstrated consistent suppression of BRCA1 levels to an average of 58% following treatment with miRNA precursors followed by MIA PaCa-2 cells, in which BRCA1 mRNA abundance was reduced to an average of 71% of control levels upon treatment (*P* < 0.01). Interestingly, hTERT-HPNE cells demonstrated the most variability in response to treatment with miR-15/107 precursors: miR-15a, miR-103a, miR-107, miR-424, and miR-497 effectively suppressed BRCA1 transcript levels to 20% compared to control transfections. On the other hand, miR-16, miR-195, and miR-503 produced comparably little suppression of BRCA1 in hTERT-HPNE cells. 293T, MDA-MB-231, and MDA-MB-468 cells consistently demonstrated weak suppression of BRCA1 mRNA levels to an average of 86%, 84%, and 96%, respectively, across miRNAs. Curiously, PANC-1 cells treated with miR-15/107 miRNAs frequently exhibited an increase in BRCA1 transcript levels with only miR-103a, miR-107, and miR-503 producing significant reductions in BRCA1 mRNA (*P* < 0.05). Although the majority of cell lines tested displayed significant decreases in BRCA1 mRNA levels following treatment with miR-15/107 miRNAs suggestive of a specific interaction, there was nonetheless appreciable variability across cell lines, perhaps as a result of cell-specific miRNA processing and off-target interactions. In all instances, a control siRNA directed against BRCA1 reduced transcript availability to <20% of control (**Figure 2**, *P* < 0.001).

### The miR-15/107 Group Targets an MRE in BRCA1’s Coding Region

**FIGURE 3:**
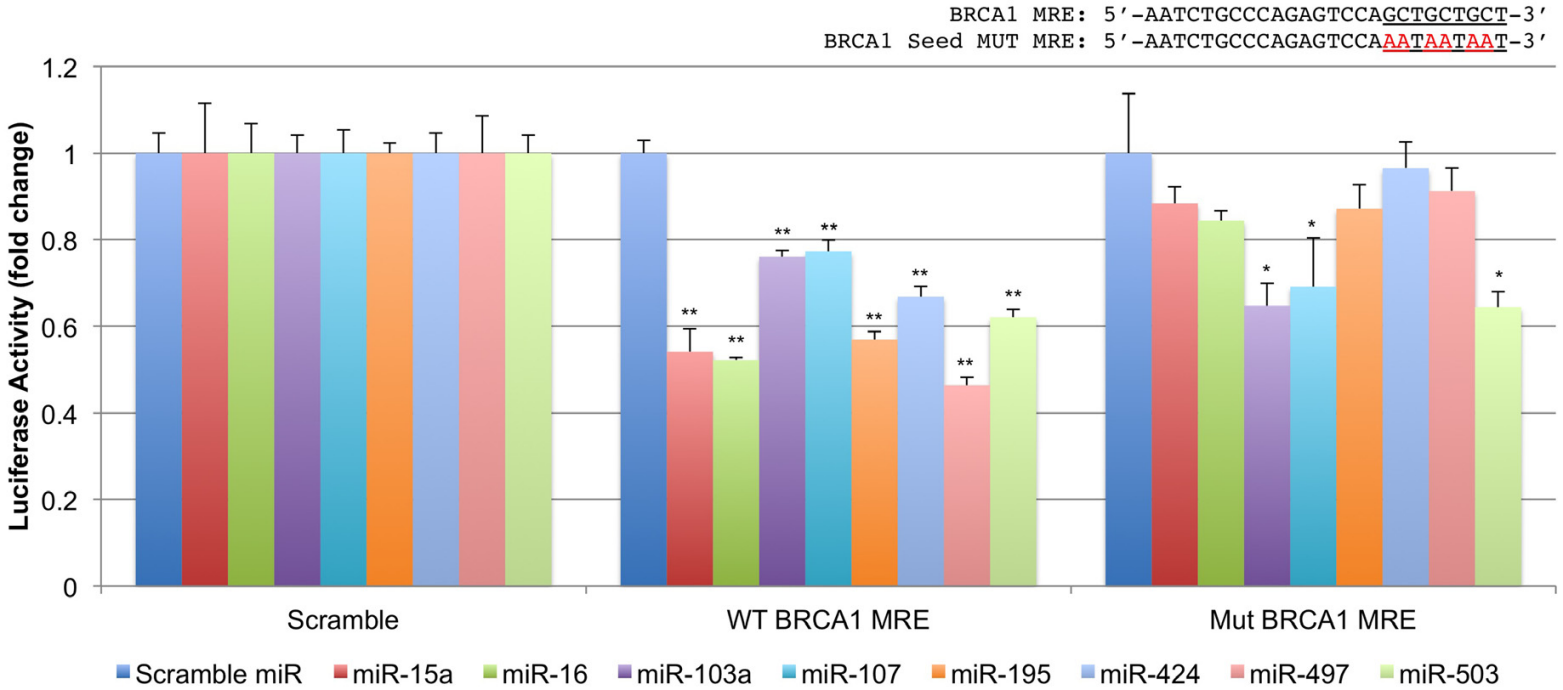
**Characterization of a novel miR-15/107 target site within the BRCA1 CDS.** The putative binding site for the miR-15/107 group in BRCA1’s CDS was cloned into a luciferase reporter vector along with a corresponding construct containing mutations within the putative miRNA ‘seed’ recognition sites to confirm specificity. ^∗^*P* < 0.05, ^∗∗^*P* < 0.01 compared to control scramble target sequence by Student’s *t*-test, *n* = 3 in each group.

We next performed luciferase target validation studies (**Figure 3**). The rna22 predicted site for the miR-15/107 group located within the BRCA1 CDS was cloned into the 3′UTR of *Renilla* luciferase and co-transfected with 50 nM of miR-15/107 precursors in 293T cells. Forty-eight hours later, all tested miR-15/107 members demonstrated significant reduction in luciferase activity in comparison to a scrambled control miRNA precursor when co-transfected with the wild-type target site (∼50%, *P* < 0.001). Interestingly, when this target sequence was mutated to abolish putative miR-15/107 seed interactions within the triple-alanine codon region (Mut BRCA1 MRE), luciferase activity was rescued in all but the miR-103a, miR-107, and miR-503 groups suggesting that these three miRNAs may exert their effects at this MRE in the absence of Watson–Crick pairing within the seed region as has been shown previously for members of this miRNA group (Nelson et al., 2011).

### Sequestration of miR-15/107 miRNAs Increases BRCA1 Transcript Availability

**FIGURE 4:**
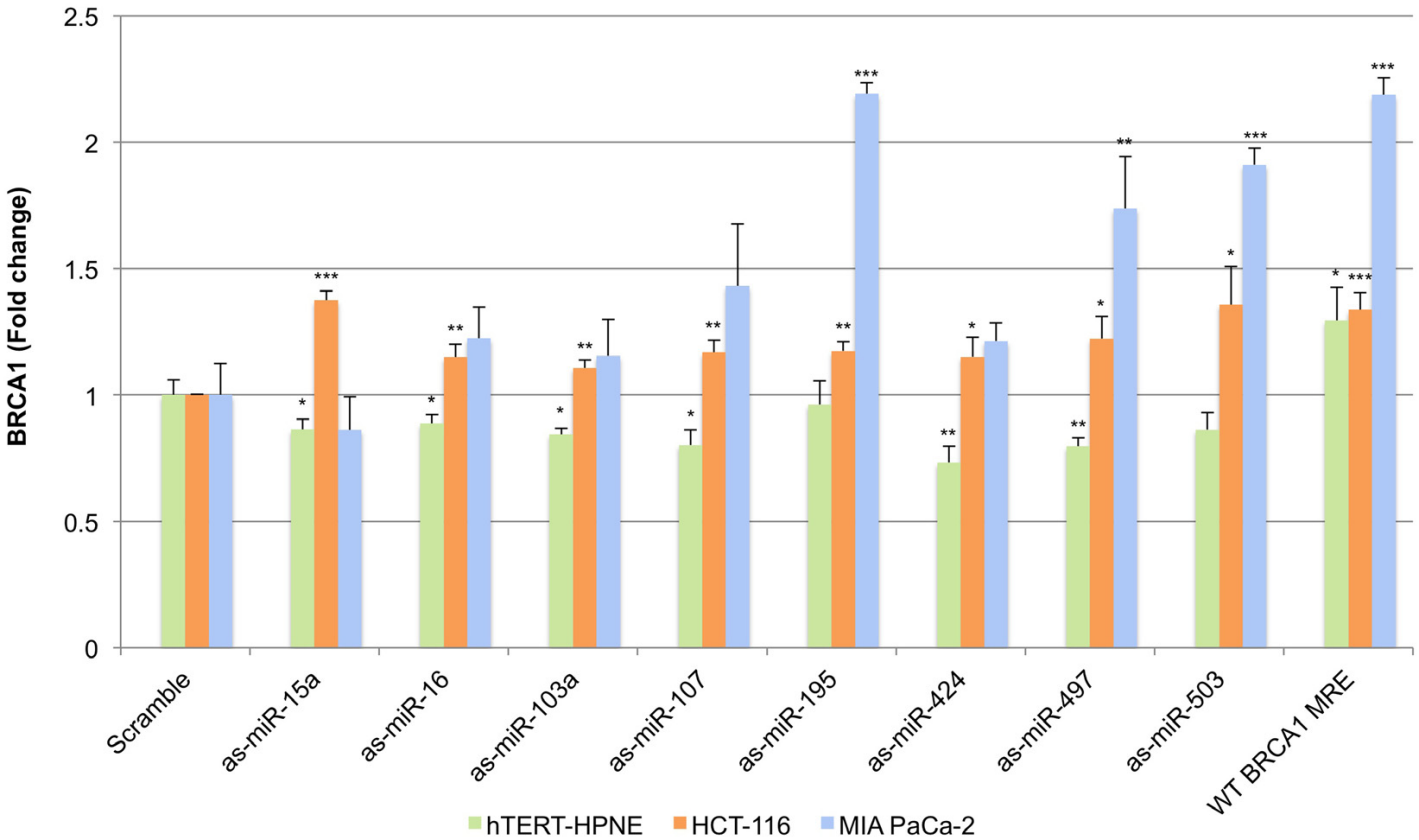
**Sequestration of miR-15/107 miRNAs impacts BRCA1 mRNA.** Cells transfected with luciferase reporter plasmids containing antisense (as) miRNA sequences or predicted wild type (WT) BRCA1 miR-15/107 MRE demonstrate varying levels of BRCA1 expression rescue. ^∗^*P* < 0.05, ^∗∗^*P* < 0.01, ^∗∗∗^*P* < 0.001 compared to control by Student’s *t*-test, *n* = 3 in each group.

We next carried out transfections with luciferase reporter plasmids containing antisense miRNA sequences as well as plasmids containing the predicted wild-type (WT) miR-15/107 BRCA1 MRE sequence inserted into the 3′UTR of *Renilla* luciferase and enumerated endogenous BRCA1 mRNA levels by qRT-PCR (**Figure 4**). For this purpose, we selected hTERT-HPNE, HCT-116, and MIA PaCa-2 cells as they demonstrated a spectrum of responses to the miR-15/107 group at the BRCA1 mRNA level ranging from moderate (MIA PaCa-2) to variable (hTERT-HPNE) to strong (HCT-116). Unexpectedly, we observed a consistent (albeit weak) *suppression* of BRCA1 levels in hTERT-HPNE cells upon transfection with the anti-miR plasmids. On the other hand, transfection with the WT MRE increased BRCA1 levels as expected. This suggests that surrogate miRNAs may exist that act upon this site in hTERT-HPNE cells and do not belong to the miR-15/107 group, or, that a complex regulatory program is in effect within these cells that involves BRCA1, the miR-15/107 group, and presumably other post-transcriptional regulators. Unlike hTERT-HPNE cells, HCT-116 cells exhibited a consistent increase of BRCA1 levels following transfection with the anti-miR plasmids and the WT MRE, as expected. The greatest rescue effect was observed in MIA PaCa-2 cells following transfection with as-miR-195, as-miR-424, and as-miR-503, in which BRCA1 levels increased between 1.7 and 2.1-fold above control transfected cells (**Figure 4**, *P* < 0.001).

### The Impact of the miR-15/107 Group on BRCA1 Protein Abundance is Specific to Cell Type

**FIGURE 5:**
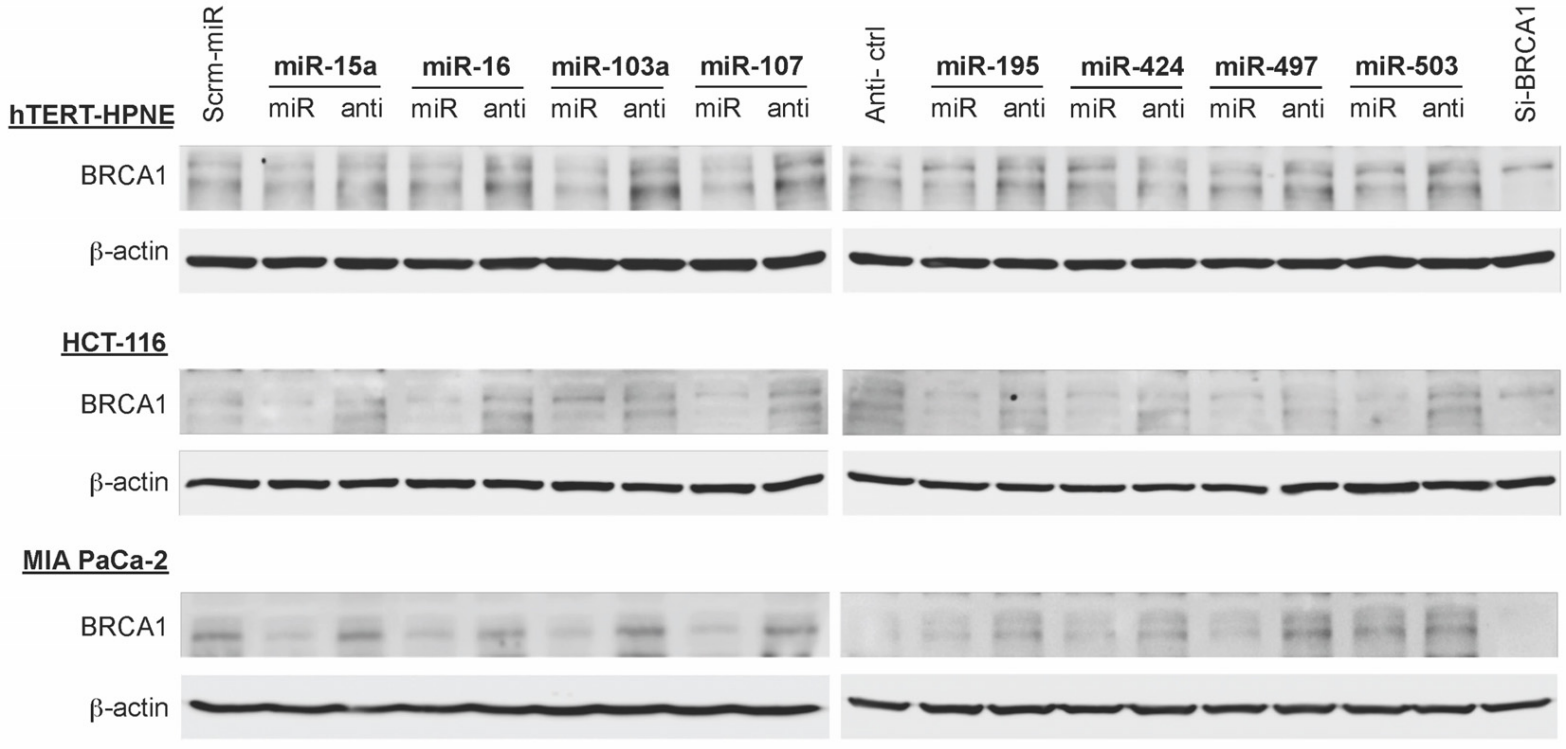
**Translational suppression of BRCA1 by miR-15/107 miRNAs.** Western immunoblots from hTERT-HPNE, HCT-116, and MIA PaCa-2 cells demonstrate relative levels of BRCA1 protein expression at 72 h post-transfection with control scramble miRNA and anti-miR precursors, miRNA and anti-miR precursors, or siBRCA1 control.

To examine the effects of the miR-15/107 group on the abundance of BRCA1 protein we performed western immunoblotting analysis of hTERT-HPNE, HCT-116, and MIA PaCa-2 cell lines transfected with miR-15/107 group members and their respective anti-miR precursors. As explained above, we selected these cell lines because they demonstrated a spectrum of responses to the miR-15/107 group at the mRNA level (moderate: MIA PaCa-2; variable: hTERT-HPNE; strong: HCT-116). We observed that in hTERT-HPNE and HCT-116 cells, treatment with miRNA precursors produced little in the way of BRCA1 protein suppression compared to their respective controls. Instead, we found appreciable increases in BRCA1 protein in these two cell lines after treatment with anti-miRs, suggesting that in these tissues there is more room for dynamic increase of BRCA1 protein following removal of miR-15/107 group members, possibly as a result of constitutive saturation of BRCA1 MRE sites by these miRNAs (**Figure 5**). Following transfection with miR-15/107 precursors, we found that MIA PaCa-2 cells demonstrated much stronger suppression of BRCA1 at the protein level compared to the suppression at the mRNA level; MIA PaCa-2 cells also showed the strongest recovery upon treatment with the anti-miRs (**Figure 5**). Across all cell lines examined, miRNA and anti-miR precursors, as well as BRCA1 siRNA controls appeared to affect the presence of detectable BRCA1 protein species in a subtle, yet differential manner according to their molecular weight. Although we did not pursue these observations further, we anticipate these differences could be due to a variety of possible sources that include one or more of the following: variations in basal expression profiles of BRCA1 mRNA splice variants, proteolytic activity and/or post-translational modification patterns across cell lines, possibly secondary to direct or indirect interactions of these RNAs.

## Discussion

Here we describe a novel target site located within a protein-coding region of the BRCA1 transcript that is common to the majority of its functional splice variants. Interestingly, this target site is conserved in higher order mammals, with only primates displaying complete sequence homology (Supplementary Figure S1; Cunningham et al., 2015). This target site is located outside of known protein coding domains that are associated with BRCA1 loss of function in human cancers when mutated, which in turn suggests the possibility of additional mechanisms through which activity of this crucial regulator of genomic integrity may be suppressed in human disease (Fackenthal and Olopade, 2007). Interestingly, members of the miR-15/107 group of miRNAs are known to be dysregulated in a number of neoplastic disorders, many of which, such as breast, ovarian, and prostate cancers, also demonstrate loss of BRCA1 function (Bhattacharya et al., 2009; Li et al., 2011; Musumeci et al., 2011; Polytarchou et al., 2012; Wang et al., 2013). Importantly, while the miR-15/107 group has been implicated in a number of cellular processes commonly disrupted in disease, namely, regulation of cell cycle, metabolism, angiogenesis, and response to cell stress (Finnerty et al., 2010), this reports highlights an additional role for this ubiquitously expressed family of miRNAs in the regulation of DNA damage repair pathways. Similarly, other groups have recently reported that these miRNAs post-transcriptionally regulate other critical DNA repair genes such as RAD51 (Huang et al., 2013).

Although there have been reports of post-transcriptional regulation of BRCA1 by other miRNAs, such as miR-182, miR-146, miR-638, and miR-17, these were all shown to exert their effects through standard interactions within the 3′UTR (Shen et al., 2008a, 2009; Garcia et al., 2011; Moskwa et al., 2011; Li et al., 2012). Of note, miR-15a and miR-16 have also been ascribed to regulate BRCA1 through a target site within the 3′UTR as treatment with anti-miRs specific to miR-15a and miR-16 results in the increased expression in a luciferase reporter system containing this target site (Zhu et al., 2009). Therefore, it stands to reason that there are likely many other points through which miRNAs of the miR-15/107 group or other miRNAs can regulate BRCA1 expression at the post-transcriptional level. This study, however, highlights the potential for regulation of BRCA1 by non-standard miRNA interactions occurring within the coding region. Further, while forced expression of miR-15/107 members resulted in suppression of BRCA1 transcript levels <80% in five different cell line models, it appears that this effect is moderated primarily by those miRNAs with the conserved AGCAGC motif located within their 5′ seed region, as mutation of the predicted seed recognition sequence within the BRCA1 MRE resulted in rescue of luciferase activity when co-transfected with these miRNAs. In contrast to this, miR-103a and miR-107, which contain an AGCAGC motif in the first six nts at their 5′ end, did not demonstrate appreciative rescue upon mutation of the putative seed recognition sequence in the BRCA1 MRE, consistent with previous reports that these miRNAs may engage in “seed-less” target selection through interactions with their 3′ end (Nelson et al., 2011). It stands that miR-503 may also be participating in “seed-less” interactions at this site as luciferase suppression was still observed following mutation of the putative seed recognition sequence in a similar manner.

Generally, we did not observe clear associations between BRCA1 protein levels and transcript availability following treatment with miRNA precursors of the miR-15/107 group. The most appreciable differences in BRCA1 protein levels were observed in MIA PaCa-2 cells, which paradoxically displayed only moderate responses at the mRNA level compared to hTERT-HPNE and HCT-116. HCT-116 and hTERT-HPNE cells demonstrated variable up-regulation of BRCA1 protein following sequestration of endogenous miRNA activity by anti-miRs. Irrespective of the mechanisms underlying these observations, our findings are consistent with recent large-scale transcriptome and proteome profiling studies of mammalian cells that indicate individual protein abundances are largely determined by post-transcriptional regulation and by translational and degradation activity rather than by copy numbers of a corresponding mRNA within a cell (Schwanhausser et al., 2011; Vogel and Marcotte, 2012; Londin et al., 2014). Such effects of post-transcriptional regulation of BRCA1 need not be unique to miRNAs as interestingly, prior to the discovery of miRNA-mediated regulation of BRCA1, the RBP HuR was shown to post-transcriptionally suppress translation of BRCA1 by associating with its 3′UTR (Saunus et al., 2008).

Experimental evidence by other groups and by us suggests that while uncommon, it may be possible for miRNAs to promote stabilization of target miRNA transcripts or induce their expression, which can result in increased downstream translation (Li et al., 2006; Miranda et al., 2006; Place et al., 2008; Huang et al., 2012). Such miRNA-induced expression of target transcripts is best characterized as occurring through interactions of miRNAs with upstream promoter regions and activation of transcription complexes and could, in part, explain some of the observed discrepancies between cell lines relating to BRCA1 transcript availability and protein concentrations (Place et al., 2008; Huang et al., 2012). While we did not investigate interactions of the miR-15/107 group and the BRCA1 promoter region in this report, they nonetheless may exist to provide yet another means for which this group of miRNAs can regulate expression of an important DDR mediator.

Taken together these results highlight the observation that the effect of miR-15/107 group miRNAs on BRCA1 is tissue-specific and suggests that miR-15/107-mediated interactions are part of molecular mechanisms with varying affinities for suppressing the abundance of BRCA1 mRNA and protein. Alternatively, it is conceivable that there are additional tissue-specific post-transcriptional regulators that are currently unknown, the combined effect of which is reflected by the abundance of BRCA1 mRNA and protein in a given tissue. In other words, our experimental observations are likely the downstream effect (at the translational level) of a multitude of endogenous molecules such as miRNA, mRNAs, RBPs, etc., whose repertoires vary in each cell line. Given the significant length of its mRNA and the fact that we have demonstrated the existence of one miRNA target in BRCA1’s CDS it is reasonable to assume that additional miRNA target sites exist along its length. These miRNAs are currently unknown and their effect is unaccounted for. An alternative interpretation of these observations is that they could be the result of low basal levels of BRCA1 protein across cell lines, or, of variations in preference for translated mRNA splicing variants, something that has been demonstrated to occur frequently in other systems (Pascal et al., 2008).

The functional significance of this non-standard regulation of BRCA1 by the miR-15/107 group remains to be elucidated. BRCA1 functional status has demonstrated itself to be of great clinical value over the past several years, as BRCA1 deficient tumors have proven to be highly susceptible to selective modulators of DNA damage repair pathways such as PARP inhibitors, and therefore can be exploited pharmacologically to effectively treat these cancers in a manner that is less cytotoxic to healthy tissues than traditional chemotherapy regimens (Helleday, 2011). As it has been shown that up-regulation of miR-182, a known miRNA modulator of BRCA1, effectively renders cells BRCA1 deficient and therefore susceptible to such PARP inhibitors as 4-amino-1,8-naphthalimide or olaparib, it may be possible that forced expression of the miR-15/107 family, which are typically down-regulated in many cancers through various mechanisms, may be advantageous in chemo-sensitizing particular cancers (Moskwa et al., 2011). As an example of this, expression of miR-15a and miR-16, which are commonly epigenetically silenced in non-small cell lung cancer, can be induced pharmacologically by treatment with HDAC inhibitors trichostatin A and sodium butyrate (Chen et al., 2013). Hence, restoration of miR-15/107 expression in tumors in which these miRNAs are down-regulated or silenced may potentially enhance response to targeted therapies such as PARP inhibitors in addition to reinstating the anti-cancer properties already attributed to these miRNAs (Bonci et al., 2008; Aqeilan et al., 2010; Finnerty et al., 2010; Furuta et al., 2013; Guo et al., 2013). However, our findings here suggest that physiological variation of miR-15/107 activity on BRCA1 across tissues may deemphasize the potential for these miRNAs as biomarkers or modulators to pharmacotherapy that targets BRCA1-mediated DSB repair. While such paradigms could be of great relevance potentially by virtue of the near-ubiquitous expression patterns of the miR-15/107 group and its members’ frequent dysregulation in disease, our tissue-specific findings suggest that a therapeutic exploitation of these interactions may not be straightforward. Indeed, it is reasonable to conjecture that the post-transcriptional regulation of high-value mRNA targets such as BRCA1 is controlled by multiple mediators such as miRNAs, RBPs, and possibly other mechanisms that are not currently known. Consequently, it is also reasonable to expect that a mistimed or uncoordinated attempt at suppression of a desired subset of targets, in this case BRCA1, may result in no observable effect, or in an effect different than what was intended. Thus, an improved understanding and a more detailed enumeration of these interactions will be required before such an intervention is attempted and further studies are warranted to explore the emerging role of non-standard miRNA mediated regulation of critical DNA damage repair factors as they pertain to the development and progression of cancers and other diseases related to genomic instability.

## Author Contributions

KQ, YJ, and IR designed the experiments. KQ and YJ performed experiments. KQ and IR wrote the manuscript. IR conceived and supervised the project. All authors have reviewed and approved the final manuscript.

## Conflict of Interest Statement

The authors declare that the research was conducted in the absence of any commercial or financial relationships that could be construed as a potential conflict of interest.

## Abbreviations

CDS: coding sequence
DDR: DNA damage response
HDR: homology directed repair
miRNA: microRNA
MRE: miRNA recognition element
mRNA: messenger RNA
nt: nucleotide
nts: nucleotides
PARP: poly ADP-ribose polymerase
qRT-PCR: quantitative reverse-transcription polymerase chain reaction
RBP: RNA binding protein
RISC: RNA induced silencing complex
SNP: single nucleotide polymorphism
UTR: untranslated region
