# Supplementary figures and images for: Post-transcriptional regulation of BRCA1 through its coding sequence by the miR-15/107 group of miRNAs
Source: Front Genet. 2015 Jul 24;6:242. doi: 10.3389/fgene.2015.00242 (PMC4513244; doi:10.3389/fgene.2015.00242)

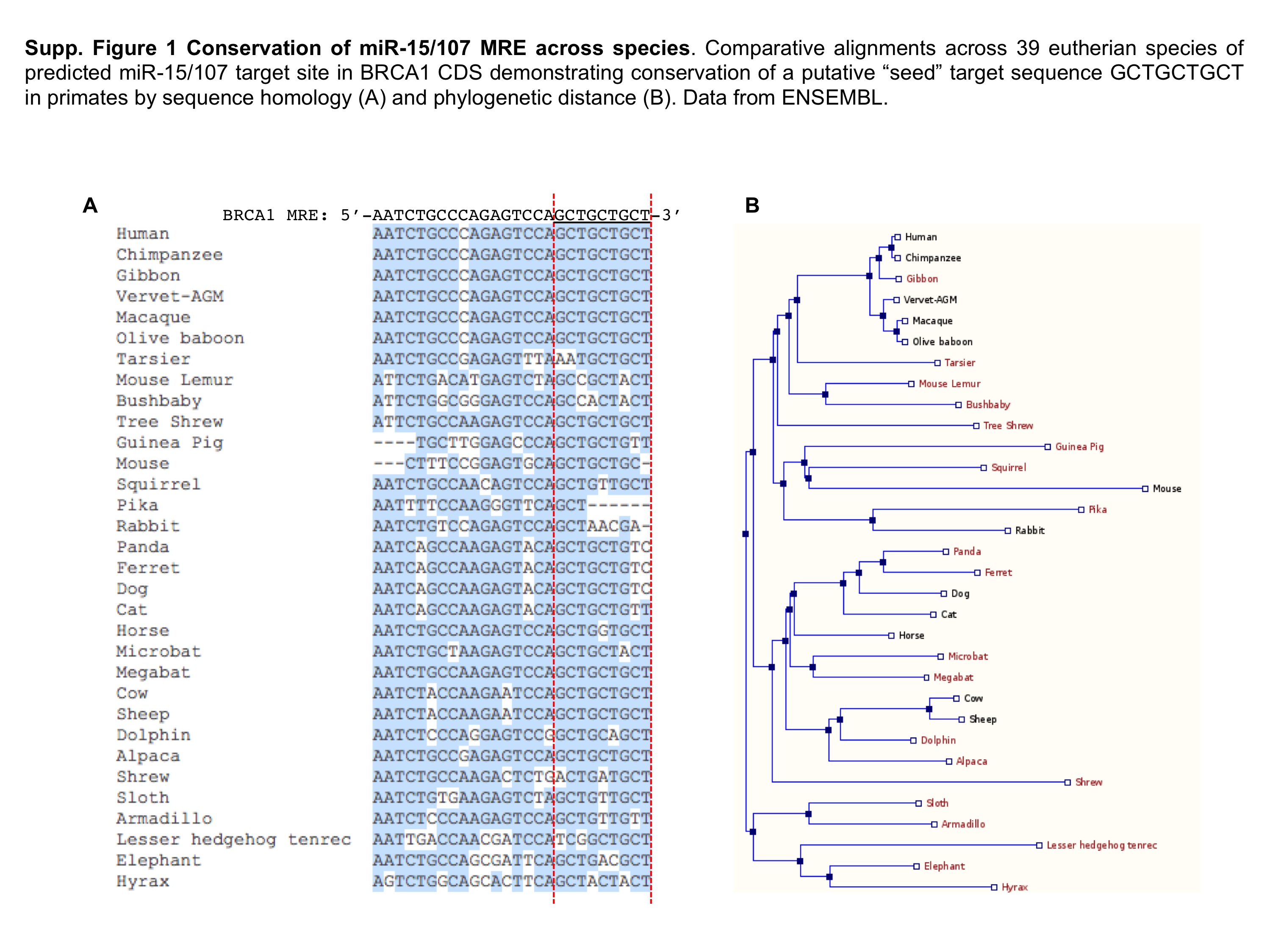

Supplement: Supplementary file 1 [file Image_1.JPEG]
